# Comparative transcriptome analysis reveals genes involved in trichome development and metabolism in tobacco

**DOI:** 10.1186/s12870-024-05265-4

**Published:** 2024-06-13

**Authors:** Mingli Chen, Zhiyuan Li, Xinxi He, Zhe Zhang, Dong Wang, Luying Cui, Minmin Xie, Zeyu Zhao, Quan Sun, Dahai Wang, Jiameng Dai, Daping Gong

**Affiliations:** 1 Tobacco Research Institute of Chinese Academy of Agricultural Sciences, Qingdao, China; 2 China Tobacco Hunan Industry Co., Ltd, Changsha, China; 3 Graduate School of the Chinese Academy of Agricultural Sciences, Beijing, China; 4 College of Bioinformation, Chongqing Key Laboratory of Big Data for Bio Intelligence, Chongqing University of Posts and Telecommunications, Chongqing, China; 5 Shandong Weifang Tobacco Co., Ltd, Weifang, China; 6 Yunnan Key Laboratory of Tobacco Chemistry, China , Tobacco Yunnan Industrial Co., Ltd, Kunming, China

**Keywords:** Glandular trichomes, Tobacco, Secondary metabolism, Development, Transcription factors

## Abstract

**Background:**

The glandular trichomes of tobacco (*Nicotiana tabacum*) can efficiently produce secondary metabolites. They act as natural bioreactors, and their natural products function to protect plants against insect-pests and pathogens and are also components of industrial chemicals. To clarify the molecular mechanisms of tobacco glandular trichome development and secondary metabolic regulation, glandular trichomes and glandless trichomes, as well as other different developmental tissues, were used for RNA sequencing and analysis.

**Results:**

By comparing glandless and glandular trichomes with other tissues, we obtained differentially expressed genes. They were obviously enriched in KEGG pathways, such as cutin, suberine, and wax biosynthesis, flavonoid and isoflavonoid biosynthesis, terpenoid biosynthesis, and plant–pathogen interaction. In particular, the expression levels of genes related to the terpenoid, flavonoid, and wax biosynthesis pathway mainly showed down-regulation in glandless trichomes, implying that they lack the capability to synthesize certain exudate compounds. Among the differentially expressed genes, 234 transcription factors were found, including AP2-ERFs, MYBs, bHLHs, WRKYs, Homeoboxes (HD-ZIP), and C2H2-ZFs. These transcription factor and genes that highly expressed in trichomes or specially expressed in GT or GLT. Following the overexpression of R2R3-MYB transcription factor Nitab4.5_0011760g0030.1 in tobacco, an increase in the number of branched glandular trichomes was observed.

**Conclusions:**

Our data provide comprehensive gene expression information at the transcriptional level and an understanding of the regulatory pathways involved in glandular trichome development and secondary metabolism.

**Supplementary Information:**

The online version contains supplementary material available at 10.1186/s12870-024-05265-4.

## Introduction

Trichomes are epidermal structures widely conserved across the plant kingdom. Trichomes are extensions of the epidermis, originating from epidermal cells, and they can be composed of a single or multiple cells. These structures are divided into two general categories: glandular or non-glandular, depending on their morphology and secretion capability. Glandular trichomes are usually multicellular and composed of three parts: a base cell in the epidermal cell layer, one or more stalk cells, and a gland with secretory cells at the apex [1]. Apical cells of glandular trichomes are unique in that they have the ability to synthesize, secrete, and store large amounts of secondary metabolites. These specialized metabolites include terpenoids, phenylpropanoids, flavonoids, methylketones, acyl sugars, and some oils or resins [2]. These natural plant compounds not only protect plants against pathogens and herbivores, but they are also components of industrial chemicals used in pesticides, food additives, and pharmaceuticals. For example, glandular trichomes of mint and basil can produced essential oils [3]. The artemisinin secreted by the glandular trichomes of *Artemisia annua* is used for the treatment of malaria [4]. Glandular trichome systems which have a high exudation capacity may be used in molecular farming to produce and exude useful biochemicals.

Tobacco (*N. tabacum*) glandular trichomes form a high-quality material for studying secondary metabolism and development [2, 5]. The glandular trichomes cover almost the entire plant, expect roots, during the entire tobacco life cycle. There are two main types of glandular trichomes on tobacco leaves, short trichomes, having unicellular stalks and multicellular heads, and tall trichomes, having multicellular stalks possessing uni- or multi-cellular heads [6]. Tobacco glandular trichomes mainly synthesize and secrete two types of diterpenoids, the polycyclic labdanoids and the macrocyclic cembranoids, which account for ~ 10% of leaf dry weight in some cultivars [7]. The labdanoid *Z*-abienol can be used as a precursor for the semi-synthesis of amber compounds used in the fragrance industry [8]. Chemical structures of cembratrien-diols (CBT-diol) may act as inhibitors of tumor-promoters, aldose reductase, and prostaglandin synthesis [9, 10].

In the biosynthesis of terpenoids, two genes encoding terpene synthase (*NtCPS2*) and kaurene synthase (*NtABS*), which are involved in the biosynthesis of the labdane diterpene *Z*-abienol, have been characterized in tobacco. *NtCPS2* encodes a class-II terpene synthase that synthesizes 8-hydroxy-copalyldiphosphate, and *NtABS* encodes a kaurene synthase-like protein that uses 8-hydroxy-copalyldiphosphate to produce bicyclic diterpene *Z*-abienol [11]. The cyclization of geranylgeranyl diphosphate by cembratrien-ol synthases leads to α- and β-cembratrien-ol, which were subsequently hydroxylated to α- and β-cembratrien-diol, respectively, by cytochrome P450 CYP71D16 [7]. In addition, *NtLTP1* has a lipid transfer function that can regulate lipid secretion from glandular trichomes [12].

With the development of sequencing technology, this method is now frequently used to study the development and metabolism of glandular trichomes. By comparing glandular trichomes with leaves through the sequencing of ESTs, proteomes, transcriptomes of tobacco glandular trichomes have been used to screen genes involved in biotic and abiotic stress responses [6, 13–15], secondary metabolism [16, 17], and gene regulatory networks associated with trichomes [18]. However, most research has focused on metabolism and rarely on the development of glandular trichomes in tobacco. While the main source of knowledge regarding trichomes is in the development of *Arabidopsis thaliana*, GLABRA3, TRANSPARENT TESTA GLABRA1, and GLABRA1 have been found to act as positive transcription factors [2]. Additionally, miRNA156 and the targeted gene SQUAMOSA PROMOTER BINDING PROTEIN LIKE 9 are involved in regulating trichome development [19]. Recently, much progress has been made in unraveling glandular trichome development in tomato and *A. annua.* Most genes regulating trichome development belong to the R2R3-MYB, HD-ZIP IV, C2H2, and bHLH-type transcription factors [20]. The R2R3-MYB proteins SlMIXTA1 and SlMixta-like control trichome initiation in tomato [21, 22]. The R2R3-MYB proteins AaMIXTA1, AaTAR2 and AaMYB16 positively regulate trichome initiation in *A. annua* [23, 24]. Conversely, AaMYB5, TLR1 and TLR2 have been found to negatively regulate trichome development in *A. annua* [24, 25]. HD-ZIP IV transcription factors SlWO and SlCD2 regulates type I and type VI glandular trichome formation in tomato, respectively [26, 27]. SIHD8 positively regulates tomato trichome elongation [28]. In *A. annua,* AaHD1 and AaHD8 positively regulate the initiation of glandular hairs [29]. Three C2H2 zinc finger proteins have been identified in tomato trichome formation. Hair (H) interacts with *SlZFP8L* to regulate trichome initiation and elongation by regulating *SlZFP6* expression in tomato [30]. The bHLH transcription factors SlMYC1 regulates the development of type VI glandular hairs in tomato [31]. Additionally, the JAZ protein SlJAZ2, SIJAZ4 and AaJAZ8 act as repressors of the JA signaling pathway, negatively regulating the occurrence of glandular hairs [28, 32, 33]. Therefore, further research on the development and regulation of glandular trichomes and secondary metabolism is needed in tobacco.

In this study, through high-throughput transcriptome sequencing combined with published tobacco genome data [34, 35], the gene expression levels of the glandless and glandular trichomes were compared to obtain key genes related to the development and metabolism of the latter. The results establish a foundation for further research on molecular mechanisms involved in tobacco glandular trichome development and secondary metabolism.

## Materials and methods

### Plant material

Tobacco varieties TI 1112 and K326 were grown in the greenhouse of the Tobacco Research Institute of the Chinese Academy of Agricultural Sciences (Qingdao) under controlled temperature (25/18 °C, day/night, winter; 32/20 °C, day/night, summer) and a natural photoperiod. Young leaf samples were harvested from three plants. The samples were immediately frozen in liquid nitrogen, and then, the trichomes from the leaf surfaces were scratched using a hairbrush. The samples were designated as glandular trichome (GT, K326) and glandless trichome (GLT, TI 1112) and were stored at − 80 °C until further use.

### Scanning electron microscopy

Trichomes on fully expanded leaves of tobacco plants were initially observed under a stereomicroscope. The type of trichome was identified by scanning electron microscopy (SEM). The SEM procedure was performed as follows: Leaves and stems were fixed in 2% glutaraldehyde, dehydrated in a graded ethanol series, sputter coated with gold, and analyzed under a JSM-6390/LV scanning electron microscope.

### Leaf surface chemistry evaluations

Eight 4 cm × 4 cm leaf disks were collected from the middle leaves of each of the three plants. Leaf disks were washed thrice for 2 s with 100 mL of HPLC grade CH_2_Cl_2_. The washings were then filtered through folded filter paper containing about 20 g of anhydrous Na_2_SO4 into round bottom flask. The solvent volume was reduced to about 0.5 ml on a rotary evaporator by heating at 40 ℃. The Gas Chromatography-Mass Spectrometry (GC–MS) analysis was conducted using an Agilent 7890A GC system with 5975C MSD (Agilent, USA). Separation was assured using an 30 m DB-5 column (0.25 mm i.d. and 0.25 μm film thickness). The samples were injected using a injector at 280 ℃. The oven was programmed to begin operating, then heat to 160 ℃ for 1 min, 260 ℃ for 2 min, 280 ℃ for 2 min, and maintain 290 ℃ for 5 min. The ionization voltage was 70 eV. These experiments were performed in triplicate.

### Transcriptome sequencing

Six trichome mRNA libraries were constructed as suggested by Illumina at Novogene (China). Briefly, total RNA was isolated using a Plant RNA Kit (TIANGEN, China) in accordance with the manufacturer’s instructions. Then, the total RNA of each sample was used for library construction. Finally, the library was sequenced using an Illumina NovaSeq 6000 (Illumina, USA).

Sequence Read Archive (SRA) entries for nine tissues, dry capsule (C), root (R), stem (S), mature flowers (MF), mature leaf (ML), senescent flower (SF), senescent leaf (SL), immature flower (IF), and young leaf (YL), were download from NCBI (PRJNA208209) for TN90 by Illumina HiSeq 2500.

### Alignment of RNA-Seq reads to the reference genome

Clean reads filtered from raw reads were mapped onto the *N. tabacum* K326 genome (ftp://ftp.solgenomics.net/genomes/Nicotiana_tabacum/edwards_et_al_2017/) using Tophat2 with default parameters [36, 37]. Gene expression levels of individual genes were quantified using Fragments Per Kilobase of transcript per Million mapped reads (FPKM) using Cufflinks 2.2.1 with default parameters [36].

### Differential gene expression analysis

Differentially expressed genes (DEGs) were identified by edgeR [38]. Significant DEGs were determined using the criteria log2|ratio|> 4 and false discovery rate ≤ 0.05. To obtain the correlated samples, we used the expression level of each gene in two samples to calculate the Pearson’s correlation coefficient, and these data were converted into a heatmap. The transcription factor genes were grouped into different classes through homology searches in the PlantTFDB database v5.0 (http://planttfdb.gao-lab.org/blast.php).

### Quantitative real-time PCR analysis (qRT-PCR)

First-strand cDNA was synthesized using *Evo M-MLV* Reverse Transcription Kit II (Agbio, China). Specific primers were designed by Primer3 using genome sequences for qRT-PCR on Applied Biosystems 7500 RT-PCR system (ABI, USA). The qRT-PCR cycling conditions were 95 °C for 2 min followed by 40 cycles of 95 °C for 5 s and 60 °C for 30 s. Actin was utilized as an internal control, and biological replicates were triplicated.

Overexpression analysis of Nitab4.5_0011760g0030.1 gene in tobacco

### Overexpression analysis of Nitab4.5_0011760g0030.1 gene in tobacco

The full length of Nitab4.5_0011760g0030.1 cDNA sequence was amplified using cloning primers, and inserted into the expression vector (pCAMBIA1300) under the control of CaMV 35S promoter. The empty vector and Nitab4.5_0011760g0030.1 overexpression vector were then transformed into tobacco wild type plants by the Agrobactericum-mediated method. To screen transgenic plants, PCR amplification of the Hygromycin Resistance gene from the genomic DNA was performed as a preliminary screening step. The positive singletons were then confirmed by measuring the Nitab4.5_0011760g0030.1 expression level using qRT-PCR. After three months of transplanting the transgenic plants into the soil, the trichome morphology of the overexpressed plant and wild type tobacco was observed and counted.

## Results

### Phenotypic characteristics of trichomes and leaf surface chemistry

**Fig. 1:**
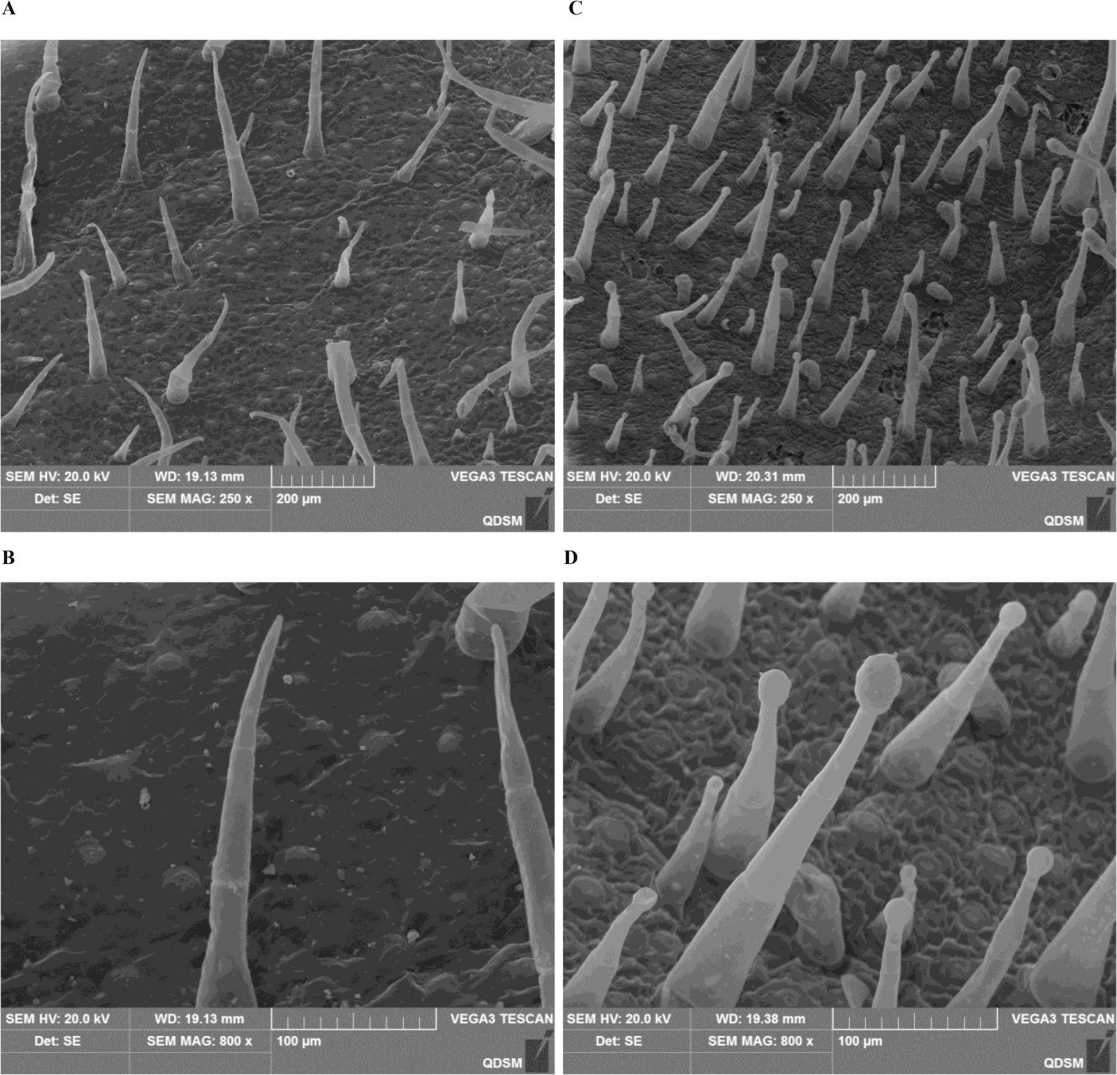
Trichomes of tobacco (N. tabacum) leaves observed using a scanning electron microscope. **A**, **B** TI 1112; (**C**, **D**) K326, Scale bars: (**A**, **C**) 200 μm and (**B**, **D**) 100 μm

Significant differences were observed between tobacco varieties TI 1112 and K326 using SEM. TI 1112 has only one type of glandless trichomes (GLT) that is mainly composed of two parts: base and sharp stalk (Fig. 1A and B). whereas K326 has very few glandless trichomes, the majority are glandular trichomes (GT) that have a uni- or multi-cellular gland at the top (Fig. 1C and D).

The GC–MS analysis was used to determine the major cuticular components of K326 and TI 1112. The glandless and glandular trichomes tobacco types produced significant differences in composition and levels of cuticular components (Figure S1). Unlike glandless trichomes, a number of surface chemical composition was observed with glandular trichomes. K326 produced a variety of duvanes, fatty alcohols, and hydrocarbon. In contrast, TI 1112 yielded very low levels of diterpenes and fatty alcohols. It appears that a few hydrocarbons, such as hentriacontane, tetratriacontane, hexatriacontane, and tetratetracontane, were not effected by trichome type.

### Differential expression gene screening of GLT and GT

**Fig. 2:**
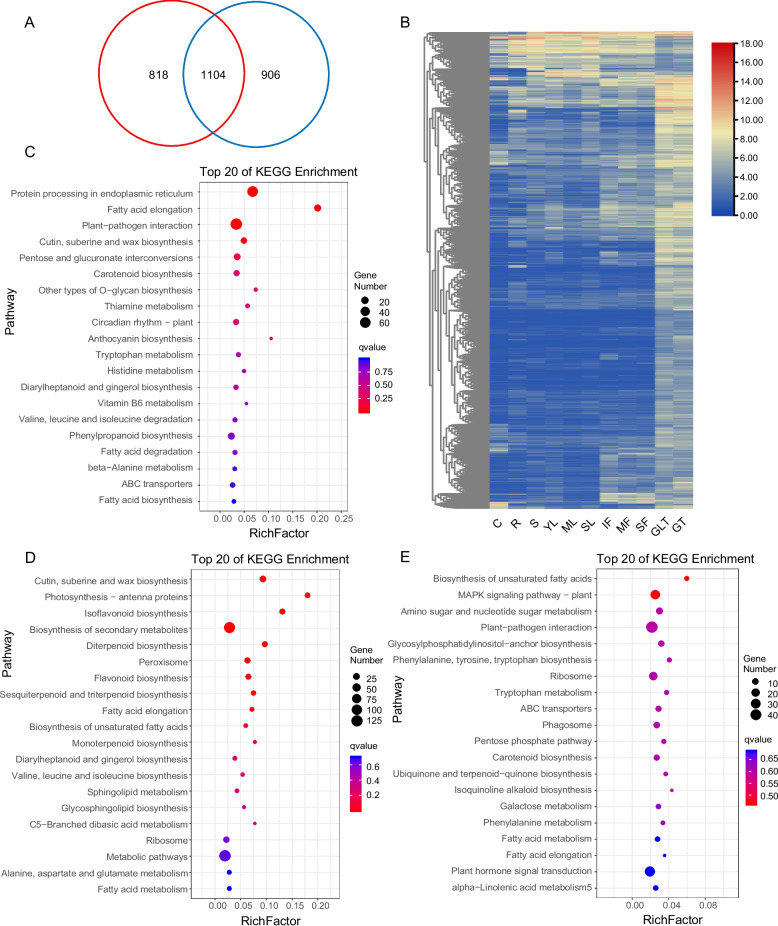
Differentially expressed gene (DEG) and functional annotations analyses of GLT and GT. (**A**) Venn diagram of tissue-specifically expressed genes in GLT and GT. **B** Heat map cluster analysis of DEGs. **C**–**E** KEGG enrichment analysis of DEGs in the intersection of the GT and GLT comparison, GT comparison group, and GLT comparison group, respectively. Dot size represents the number of distinct genes, and dot color reflects the q-value

To investigate genes involved in trichome development and metabolism between GLT and GT, six cDNA libraries were constructed for RNA Sequencing (RNA-Seq) analysis. All the transcriptional data were deposited in the NCBI Sequence Read Archive (BioProject: PRJNA786372). After filtering raw data, the six libraries produced 41.29 Gb of clean data, with Q30 and GC percentages of 92.41% and 44.19%, respectively (Table S1). The RNA-Seq was of high quality and suitable for further analyses. The clean reads were aligned to the *N. tabacum* reference genome with a cutoff FPKM as 1. To obtain special gene information related to trichome development and metabolism, the expression levels of genes in GLT and GT compared with other samples, such as dry capsule, root, stem, flower, and leaf, were compared. There were between 5,464 and 7,468 DEGs in all the compared groups. We further screened the intersection of the DEGs in GLT and GT. There were 1,922 and 2,010 common DEGs in all GLT-related comparison groups and GT-related comparison groups, respectively (Fig. 2A). These genes may mainly relate to the development and metabolism of glandular trichomes. Because the phenotypes of the GLT and GT were obviously different, the DEGs between GLT and GT were further compared. There were 1,104 common DEGs among all the comparison groups of GT and GLT, 818 DEGs that only appeared in the GT comparison group, and 906 DEGs that appeared only in the GLT comparison group.

### The highly or specifically expressed genes in GLT and GT

**Fig. 3:**
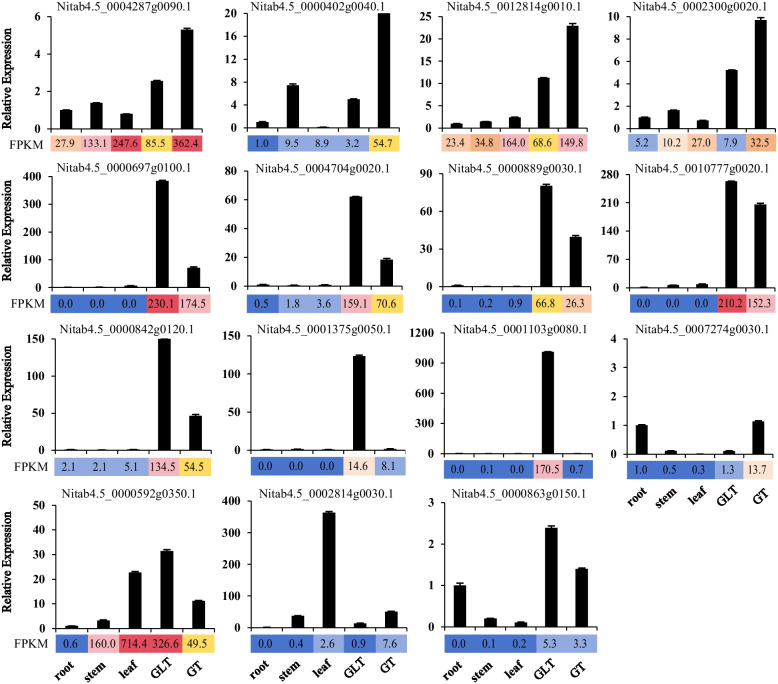
QRT-PCR confirmation of 15 DEGs. For each gene, the relative expression was calculated by setting control root as 1

As mentioned above, there were 2,828 significant DEGs between glands and other tissues (Fig. 2B). The DEGs were subject to an enrichment analysis, and 1,038 genes were mapped. These genes were mainly closely related to the biosynthesis of unsaturated fatty acids, fatty acid elongation, cutin, suberine and wax biosynthesis, isoflavonoid biosynthesis, diterpenoid biosynthesis, and plant–pathogen interactions (Table 1). To validate the expression patterns of DEGs, 15 genes were selected and analyzed by quantitative real-time reverse-transcription PCR (qRT-PCR) (Table S2). The results confirmed the accuracy and repeatability of the RNA-Seq results (Fig. 3).

**Table 1:** The KEGG pathway enrichment list

#	Pathway	Candidate genes	All genes	*P*-value	*Q*-value	Pathway ID
1	Fatty acid elongation	26 (2.5%)	85 (0.4%)	0.000000	0.000000	ko00062
2	Cutin, suberine and wax biosynthesis	41 (3.95%)	249 (1.17%)	0.000000	0.000000	ko00073
3	Photosynthesis—antenna proteins	18 (1.73%)	78 (0.37%)	0.000000	0.000001	ko00196
4	Plant-pathogen interaction	135 (13.01%)	1968 (9.24%)	0.000026	0.000580	ko04626
5	Isoflavonoid biosynthesis	18 (1.73%)	123 (0.58%)	0.000029	0.000580	ko00943
6	Biosynthesis of unsaturated fatty acids	11 (1.06%)	60 (0.28%)	0.000133	0.002312	ko01040
7	Peroxisome	27 (2.6%)	272 (1.28%)	0.000378	0.005760	ko04146
8	Diterpenoid biosynthesis	18 (1.73%)	166 (0.78%)	0.001257	0.017043	ko00904
9	Biosynthesis of secondary metabolites	283 (27.26%)	4987 (23.41%)	0.001698	0.020720	ko01110
10	Flavonoid biosynthesis	23 (2.22%)	250 (1.17%)	0.002678	0.029700	ko00941
11	Sesquiterpenoid and triterpenoid biosynthesis	13 (1.25%)	122 (0.57%)	0.006534	0.066425	ko00909
12	Limonene and pinene degradation	4 (0.39%)	18 (0.08%)	0.009907	0.092976	ko00903
13	Carotenoid biosynthesis	24 (2.31%)	302 (1.42%)	0.013083	0.114008	ko00906
14	Fatty acid metabolism	19 (1.83%)	230 (1.08%)	0.017607	0.143206	ko01212
15	Circadian rhythm—plant	23 (2.22%)	310 (1.45%)	0.030276	0.217684	ko04712
16	Tryptophan metabolism	13 (1.25%)	149 (0.7%)	0.030333	0.217684	ko00380
17	Stilbenoid, diarylheptanoid and gingerol biosynthesis	15 (1.45%)	185 (0.87%)	0.037212	0.252217	ko00945
18	MAPK signaling pathway—plant	70 (6.74%)	1166 (5.47%)	0.040894	0.262584	ko04016

The 1,104 DEGs in the intersection of the GT and GLT comparison were subjected to a Gene Ontology (GO) annotation. The results showed that in biological process, they were mainly enriched in response to biotic stimulus (GO:0009607), fatty acid biosynthetic process (GO:0006633), monocarboxylic acid biosynthetic process (GO:0072330), and response to stress (GO:0006950) GO terms. In cellular component, the DEGs were mainly enriched in cell wall (GO:0005618), external encapsulating structure (GO:0030312), and cell periphery (GO:0071944). In molecular function, the main enriched GO terms were nucleic acid binding transcription factor activity (GO:0001071) and transcription factor activity, sequence-specific DNA binding (GO:0003700). These 1,104 DEGs were also subjected to a KEGG annotation enrichment analysis. These DEGs were mainly enriched in protein processing in endoplasmic reticulum, fatty acid elongation, plant–pathogen interaction, cutin, duberine, and wax biosynthesis, and diterpenoid biosynthesis (Fig. 2C).

A GO annotation analysis of 818 unique DEGs in the GT comparison group revealed that the terpene synthase activity (GO:0010333), carbon − oxygen lyase activity, acting on phosphates (GO:0016838), and iron ion binding (GO:0005506) molecular function terms were significantly enriched. Furthermore, fatty-acyl-CoA reductase (alcohol-forming) activity (GO:0080019) and O-methyltransferase activity (GO:0008171) were relatively highly enriched terms. A KEGG annotation of these 818 DEGs revealed that cutin, suberine and wax biosynthesis, Photosynthesis-antenna proteins, isoflavonoid biosynthesis, diterpenoid biosynthesis, and flavonoid biosynthesis were enriched pathways (Fig. 2D).

A GO annotation analysis of 906 unique DEGs in the GLT comparison group revealed that the protein serine/threonine phosphatase activity (GO:0003700), protein serine/threonine phosphatase activity (GO:0004722), nucleic acid binding transcription factor activity (GO:0001071), and protein tyrosine kinase activity (GO:0004713) molecular function terms were significantly enriched. A KEGG annotation of these 906 DEGs revealed that biosynthesis of unsaturated fatty acids and MAPK signaling were enriched pathways (Fig. 2E).

### Cutin, suberine, and wax biosynthesis

**Fig. 4:**
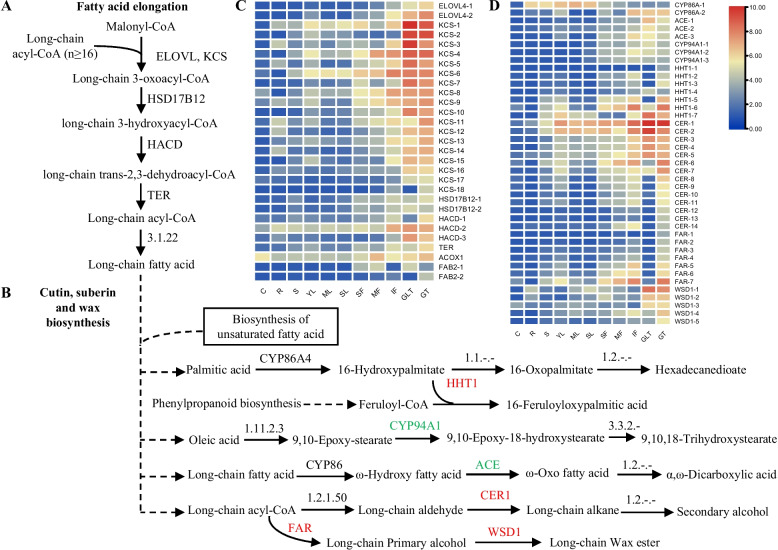
The genes involved in the cutin, suberine, and wax biosynthesis pathway. **A** Fatty acid elongation pathway. **B** Cutin, suberine, and wax biosynthesis pathway. **C** The expression patterns of genes in the fatty acid elongation pathway. **D** The expression patterns of genes in the cutin, suberine, and wax biosynthesis pathway. *ELOVL4*, elongation of very long-chain fatty acid protein 4; *KCS*, 3-ketoacyl-CoA synthase; *HSD17B12*, hydroxysteroid 17-beta dehydrogenase 12/ very long-chain 3-oxoacyl-CoA reductase; *TER*, very long-chain enoyl-CoA reductase; *HACD*, very long-chain (3R)-3-hydroxyacyl-CoA dehydratase; *ACOX*, acyl-CoA oxidase; *FAB2*, acyl-[acyl-carrier-protein] desaturase; *CYP86A*, fatty acid omega-hydroxylase; *ACE*, fatty acid omega-hydroxy dehydrogenase; *CYP94A1*, fatty acid omega-hydroxylase; *HHT1*, omega-hydroxypalmitate O-feruloyl transferase; *CER*, aldehyde decarbonylase; *FAR*, alcohol-forming fatty acyl-CoA reductase; *WSD1*, wax-ester synthase

Very long-chain fatty acids and their derivatives are the main compounds of cuticular waxes in tobacco [39, 40]. According to the KEGG enrichment analysis results, the process of fatty acid elongation, biosynthesis of unsaturated fatty acids, and cutin, suberine, wax biosynthesis were obviously enriched in GLT and GT (Fig. 4 and Table 1). The biosynthesis of unsaturated fatty acids and fatty acid elongation pathway are upstream of cutin, suberine, and wax biosynthesis, as they supply the substrate for the biosynthesis of the cutin, suberine, and wax (Fig. 4A and B). In the fatty acid elongation pathway, 2 elongation of very long-chain fatty acid proteins, 18 3-ketoacyl-CoA synthases, 2 very long-chain 3-oxoacyl-CoA reductases, 3 very long-chain (3R)-3-hydroxyacyl-CoA dehydratase, and 1 very long-chain enoyl-CoA reductase were identified (Fig. 4A). In addition, one acyl-CoA oxidase and two acyl-[acyl-carrier-protein] desaturases were identified in the biosynthesis of unsaturated fatty acids. The regulated genes in these pathways were mostly expressed in high amounts in GLT and GT, but their expression levels were relatively low in other tissues, indicating that the accumulation of fatty acids and their derivatives may be the main outcomes of the metabolism and synthesis of trichomes (Fig. 4C). In addition, many genes showed the trend of gradually decreasing as flower tissues senesced.

In the cutin, suberine, and wax biosynthesis pathway, two fatty acid omega-hydroxylases, three fatty acid omega-hydroxy dehydrogenases, three fatty acid omega-hydroxylases, seven omega-hydroxypalmitate O-feruloyl transferases (HHT1s), 14 aldehyde decarbonylases (CERs), seven alcohol-forming fatty acyl-CoA reductases, and five wax-ester synthases (WSD1s) were identified (Fig. 4B). The expressions of five *HHT1* genes were enhanced in GT, whereas the expressions of the other two *HHT1* genes were repressed. Five detected putative *CER* genes were all highly expressed at the transcript level in GT and GLT, with similar regulatory models. However, the expression levels of the remaining nine *CER* genes showed varied expression at the transcript level, with only *CER-7* being expressed in GLT. Seven genes encoding alcohol-forming fatty acyl-CoA reductases involved in wax ester biosynthesis showed greater transcript abundances in GT than GLT. Wax-ester syntheses are required in the downstream reaction of wax-ester processes in tobacco. Three *WSD1* genes with similar expression levels between GL and GLT were identified, whereas the other two *WSD1* genes showed repressed expression levels in GLT compared with in GT. In addition, three *ACE* genes and three *CYP94A1* genes with low transcript expression levels showed enhanced expressions in GLT compared with in GT. Moreover, many genes showed a decreasing expression trend in senescent flowers (Fig. 4D).

### Flavonoid and isoflavonoid biosynthesis

**Fig. 5:**
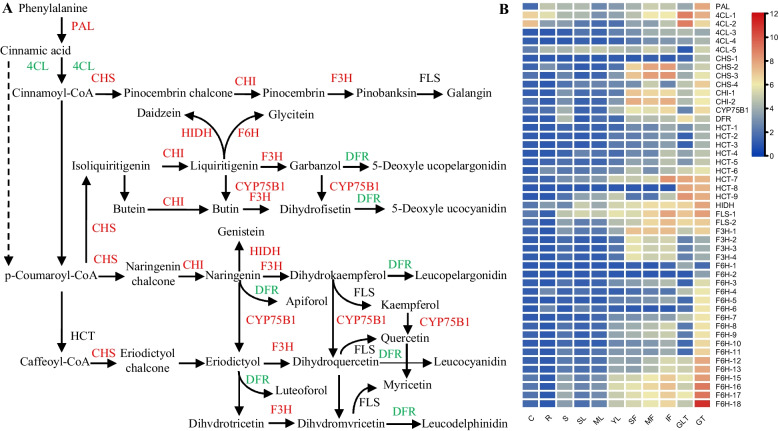
The genes involved in the flavonoid and isoflavonoid pathway (**A**) and the expression patterns in compared tissues (**B**). Gene expression is displayed in heatmaps based on mean FPKM. *PAL*, phenylalanine ammonia lyase; *4CL*, 4-coumaroyl CoA ligase; *CHS*, chalcone synthase; *CHI*, chalcone isomerase; *HCT*, shikimate O-hydroxycinnamoyltransferase; *HIDH*, 2-hydroxyisoflavanone dehydratase; *F3H*, naringenin 3-dioxygenase; *DFR*, dihydroflavonol-4-reducatse; *F6H*, flavonoid 6’-hydroxylase; *FLS*, flavonol synthase

In the biosynthesis of flavonoids, the synthesis of cinnamoyl-CoA is mainly catalyzed by two genes, phenylalanine ammonia-lyase (*PAL*) and 4-coumarate–CoA ligase (*4CL*), which is synthesized from phenylalanine. Here, the key node gene *PAL* was up-regulated, while *4CL* was down-regulated in GT. A total of 23 and 19 genes were mapped to the flavonoid and isoflavonoid biosynthesis pathways, respectively (Fig. 5A). In the flavonoid pathway, the expression levels of four chalcone synthases (CHSs), two chalcone isomerases (CHIs), four naringenin 3-dioxygenases (F3Hs), one flavonoid 3'-monooxygenase (CYP75B1), and five shikimate O-hydroxycinnamoyltransferase (HCTs) were up-regulated in GT, but not DFR, which may lead to increases in the levels of their downstream products (Fig. 4B). The two intermediate products in this pathway, naringenin and liquritigenin, can directly enter the isoflavonoid pathway. The expression levels of multiple homologous *HIDH* and *F6H* genes in GT are also up-regulated; consequently, the contents of daidzein, glycitein, genistein, and subsequent metabolites, in GT should be higher than in GLT (Fig. 5B). Except for in the GT tissues, the expression levels of multiple *CHS*, *CHI*, *HCT*, *HIDH*, *FLS*, *F6H*, and *F3H* genes were higher in flower (IF, MF, and SF) than in other tissues. Because *CHS*, *CHI*, and *F3H* genes are closely related to the synthesis of anthocyanidin, their expression levels are in line with expectations.

### Terpenoid backbone biosynthesis

**Fig. 6:**
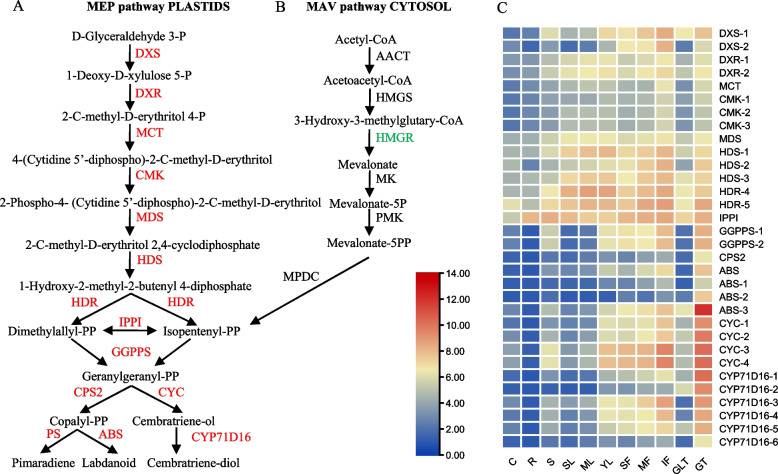
The DEGs enriched in the diterpene biosynthesis pathway (**A**) and their expression patterns (**B**). Gene names that are green and red indicate down- and up-regulation, respectively. Orange indicates that there are multiple genes which were up- and down-regulated. *DXS*, 1-deoxy-D-xylulose-5-phosphate synthase; *DXR*, 1-deoxy-D-xylulose-5-phosphate reductoisomerase; *MCT*, 2-C-methyl-D-erythritol-4-phosphate cytidylyltransferase; *CMK*, 4-cytidine 5’-diphospho-2-C-methyl-D-erythritol kinase; *MDS*, 2-C-methyl-D-erythritol-2,4-cyclodiphosphate synthase; *HDS*, hydroxy-2-methyl-2-(E)-butenyl 4-diphosphate synthase; *HDR*, hydroxy-2-methyl-2-(E)-butenyl 4-diphosphate reductase. *AACT*, acetyl-CoA C-acetyltransferase; *HMGS*, 3-hydroxy-3-methyl-glutaryl coenzyme A synthase; *HMGR*, 3-hydroxy-3-methyl-glutaryl coenzyme A reductase; *MK*, mevalonate kinase; *PMK*, phosphomevalonate kinase; *MPDC*, mevalonate diphosphate decarboxylase; *IPPI*, isopentenyl diphosphate isomerase; *GGPPS*, geranylgeranyl diphosphate synthetase; *CPS2*, copalyl diphosphate synthase; *PS*, pimaradiene synthase; *ABS*, abienol synthase; *CYC*, cembretriene-ol cyclase; *CYP71D16*, cytochrome P450

Terpenes are the most abundant compounds synthesized in plant trichomes and are the main focus in trichome metabolism research. The main terpene products are monoterpene and sesquiterpene, derived from the plastidic methylerythritol phosphate (MEP) and the mevalonate (MVA) pathways, respectively. However, the enrichment of DEGs between GLT and GT showed that they were mainly enriched in the MEP pathway (Fig. 6A and B). Multiple node genes all showed down-regulated expression levels in GLT, which reduces the production of isopentenyl-PP and dimethylallyl-PP by reducing the production of 1-hydroxy-2-methyl-2-butenyl 4-diphosphate. However, in GT, the increase in terpenoid metabolites mainly lead to the synthesis of more isopentenyl-PP and dimethylallyl-PP through the MEP pathway, resulting in their output. A MEP analysis showed that the expression levels of these genes in GLT were significantly lower than those in GT. However, the expression levels in other tissues varied greatly, revealing that these genes were not specifically expressed in glandular hairs (Fig. 6C).

#### Plant–pathogen interaction

**Fig. 7:**
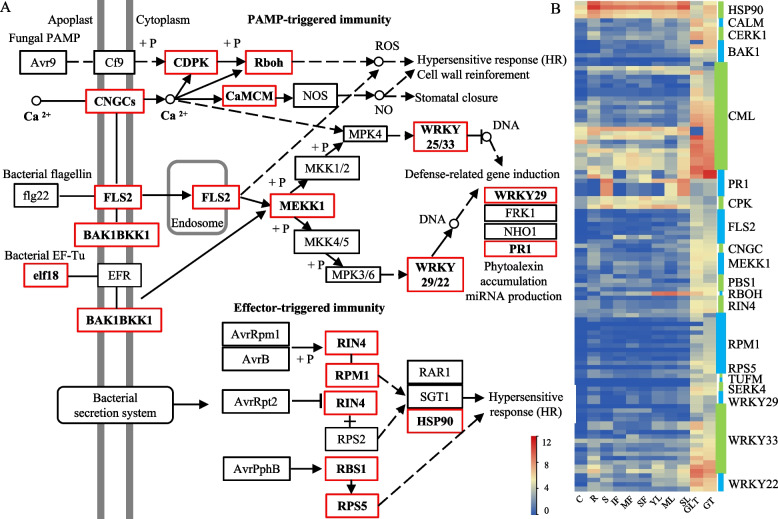
The DEGs enriched in the plant–pathogen interaction pathway (**A**) and their expression patterns (**B**). CDPK, calcium-dependent protein kinase; CALM, calmodulin. CML, calcium-binding protein; PR, pathogenesis-related protein; BAK, brassinosteroid insensitive 1-associated receptor kinase; SERK, somatic embryogenesis receptor kinase; FLS2, LRR receptor-like serine/threonine-protein kinase; CERK, chitin elicitor receptor kinase; RBOH, respiratory burst oxidase; HSP90, molecular chaperone HtpG; CNGC, cyclic nucleotide-gated channels; Rboh, respiratory burst oxidase homolog; MEKK, mitogen-activated protein kinases; RPM, resistance to pseudomonas maculicola; RIN, RPM1-interacting protein; RPS5, disease resistance protein

Trichomes are a protective barrier against pathogen attacks that use two strategies, PAMP-triggered immunity and effector-triggered immunity [41–43]. In total, 132 genes were enriched in KEGG terms related to Plant–pathogen interaction pathways, including those encoding proteins involved in pathogen-associated molecular patterns (PAMPs) perception, Ca^2+^ signaling, MAPK–WRKY signaling, and hypersensitive response, as well as pathogen-secreted proteins and disease resistance proteins. Of these, 120 genes exhibited higher expression levels in GLT or GT than in other tissues, whereas the remaining 12 genes, encoding three calcium-dependent protein kinases, four pathogenesis-related proteins, one respiratory burst oxidase homolog, and four molecular chaperone HtpGs, had lower expression levels (Fig. 7). Among those genes, two cyclic nucleotide-gated channels are involved in the regulation of the transient calcium (Ca2 +) influx into the cytosol upon PAMP/DAMP perception, three calcium-dependent protein kinases directly phosphorylate and regulate respiratory burst oxidase homolog for synthesizing ROS in PAMP-triggered immunity, and 26 genes encoding CALM/CML proteins are calcium receptor-activating enzymes and non-enzymatic proteins. Moreover, there are eight genes encoding FLS2, four genes encoding BAK1, two genes encoding SERK4, and five genes encoding mitogen-activated protein kinases, and those genes were specifically induced, or had significantly higher expression levels, in GLT and GT, except one BAK1 gene that was just expressed in GT. Thus, the trichomes of tobacco play central roles in the MAPK signal transduction pathways during responses to abiotic stresses, such as pathogen attack. In addition, WRKY proteins can act as positive or negative regulators of defense responses. In this study, 22 *NtWRKY* genes (16 *WRKY33*, 4 *WRKY22*, and 2 *WRKY29* genes) were identified from GLT and GT. The transcript levels of *NtWRKY* genes were higher in trichomes than in other tissues, such as flower, leaf, and stem. In tobacco, 4 *HSP90*s in GLT and GT are required for the defense-mediated responses of several *R* genes, including 14 resistances to *Pseudomonas syringae pv. maculicola* 1 and 4 resistances to *P. s. pv. maculicola* 1-interacting protein 4, as well as the disease resistance protein Ribosomal Protein S5). All *HSP90* genes had lower expression levels in GLT and GT, whereas the 19 *R* genes exhibited higher expression levels.

### Transcriptional regulators and relevant genes involved in trichome development

**Fig. 8:**
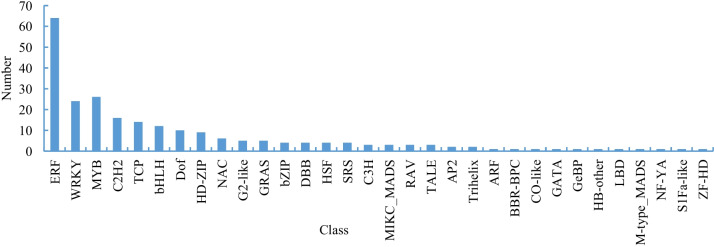
Transcription factors among the differentially expressed genes of GLT:GT

Based on a comparative transcriptome analysis, 234 differentially expressed transcription factors, which mainly including ERFs, MYBs, basic helix-loop-helixes (bHLHs), WRKYs, TCPs, Homeoboxes (HD-ZIPs), and C2H2-ZFs, were identified in this study (Fig. 8). These included 26 MYBs, 12 bHLHs, 14 C2H2-ZFs and 9 HD-ZIP transcription factors. An analysis of the expression levels of these genes revealed that three MYB transcription factors (Nitab4.5_0000842g0120.1, Nitab4.5_0004704g0020.1 and Nitab4.5_0005359g0030.1) were more highly expressed in GLT than in GT (Fig. 3). However, one member was almost exclusively highly expressed in GT. There are 12 bHLH transcription factors that are expressed at higher levels in GLT compared to GT, while their expression levels in other tissues are very low. Of the nine transcription factors in the HD-ZIP category, three members belong to HD-ZIP IV subfamily. Two HD-ZIP IV members (Nitab4.5_0002049g0010.1 and Nitab4.5_0000143g0600.1) were more highly expressed in GT than in GLT and other tissues.

The B-type cyclin genes (*cycB2*) play important roles in trichome development. In this study, four *cycB2* genes were highly expressed in GLT and GT, with the expression levels of three being higher in GL than in GLT. Phytohormones are among the major regulators of plant development, including jasmonate (JA), a crucial signaling molecule that regulates trichome development. The JA ZIM domain (JAZ) proteins act as transcriptional repressors of the JA hormonal response. Here, 12 JAZ proteins were found to be highly expressed in GLT and GT.

### Overexpression of *Nitab4.5_0011760g0030.1* gene increased the number of branched trichomes in tobacco

**Fig. 9:**
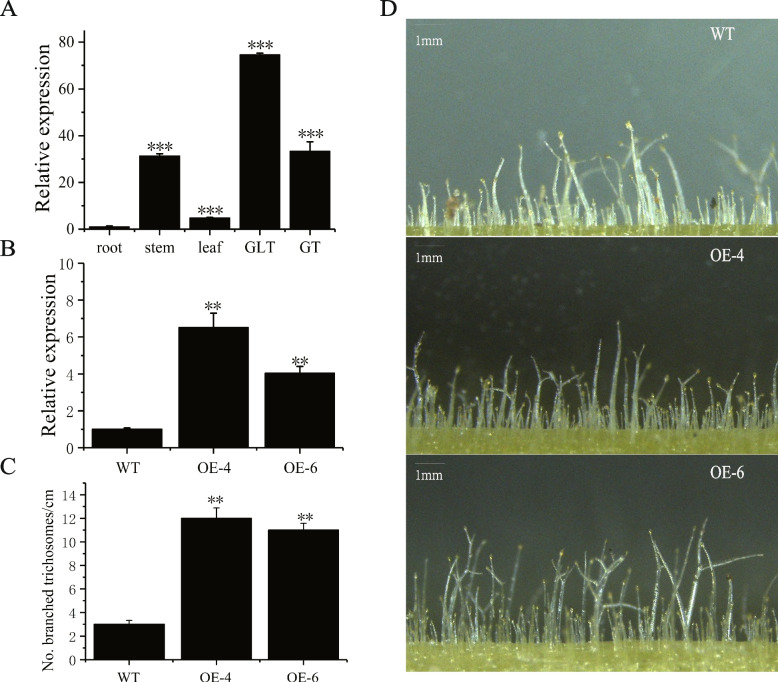
Nitab4.5_0011760g0030.1 regulate trichomes branches. **A** Expression analysis of Nitab4.5_0011760g0030.1 in different tissues. **B** Relative expression of *Nitab4.5_0011760g0030.1*. **C** Number of branched trichosomes/cm. **D** Glandular trichomes phenotype of wild type plant and transgenic plants

To further explore the potential genes regulating the development of tobacco trichomes, Nitab4.5_0011760g0030.1, a MYB transcript factor, were overexpressed in tobacco. The results of qRT-PCR analysis showed that Nitab4.5_0011760g0030.1 was highly expressed in GT and GLT (Fig. 9A). The expression levels of Nitab4.5_0011760g0030.1 gene were examined by qRT-PCR analysis in transgenic lines and the wild type tobacco. Compared to the wild type tobacco, the expression of the Nitab4.5_0011760g0030.1 was dramatically enhanced in transgenic tobaccos (Fig. 9B). Additionally, the number of branched trichomes increased significantly in transgenic lines compared to the wild type tobacco plants (Fig. 9C and D). This indicates that the Nitab4.5_0011760g0030.1 gene is involved in the development of tobacco trichomes.

## Discussion

The GLTs and GTs serve as barriers against various external factors, including UV-B radiation, extreme temperatures, herbivores, and pathogens, as well as excessive water loss [2]. High-throughput sequencing methods have been used to study GTs, but they mainly compared the gene expression levels, protein contents, and metabolites of GTs on leaves with those that had been removed [16, 44]. The comparison of these tissues inevitably includes the expression levels of some non-glandular genes, resulting in false positives in the screening results. Here, we used a GLT mutant that did not have a GT head. In the comparison of the mutant with the GT, we were most concerned with two types of genes, those related to the GT secondary metabolic network and those related to GT development.

The GTs of tobacco secretes large quantities of resin that is typically composed of a mixture of diterpenes, sucrose esters, fatty alcohols, and wax esters. In tobacco, two types of diterpenes, cembranoids and labdanoids, are generally the major cuticular components [10, 45]. Two major cembranoid diterpenes (α- and β-cembratrien-diol) account for 60% of exudates weight, together with generally smaller quantities of their respective precursors, α- and β-cembratrien-ol (CBT-ols). The major labdanoids are *Z*-abienol and labdene-diol [8]. In general, the MVA pathway is involved in the biosynthesis of sesqui-, tri-, and polyterpenes, and the MEP pathway is involved in the synthesis of mono-, di-, and tetraterpenes. In this study, the expression levels of all the genes in the MEP pathway were up-regulated in GT compared with GLT, suggesting that the synthesis of isopentenyl-pp and its subsequent metabolites may be mainly derived from the MEP pathway in GT. These results are similar to those of GTs and filamentous trichomes in *A. annua*, in which the gene expression levels in the MEP pathway are up-regulated in GTs [46]. The GLTs only produced small amounts of cembratrien-diol, fatty alcohol, and wax esters. Therefore, in this study, the down-regulation of multiple genes in GLT may lead to a reduction in diterpenes. Four reported downstream gene families, *NtCPS2* and *NtABS*, involved in the biosynthesis of the labdane diterpene *Z*-abienol, and cembratrien-ol synthase and cytochrome P450, involved in the biosynthesis of the cembranoid diterpene cembratrien-diol, have been characterized in tobacco and were confirmed here to be down-regulated in GLT [7, 11].

A series of C16–C30 fatty alcohols, C25–C36 saturated hydrocarbons, and C30–C52 wax esters have been isolated from the cuticular waxes of green tobaccos [39, 40]. The wax esters consist of alcohols bound to C12–C30 saturated fatty acids. The major alcohol is 1-docosanol, and the major acid constituent is myristic acid. In our study, the content of 1-docosanol, thunbergol and heptatriacontanol in GLTs was far lower than in GLs. The comparative transcriptome analysis of the tobacco trichomes revealed an enhanced biosynthesis of wax in GTs (Fig. 4). In the two upstream pathways, fatty acid elongation and biosynthesis of unsaturated fatty acids, few DEGs with lower expression levels were identified. The genes for the biosynthesis of long-chain fatty acids may be producing different substrates for the biosynthesis of cutin, wax, and suberin. In the wax biosynthesis pathway, *FAR* plays a key role as a fatty acyl-coenzyme A reductase that generates primary alcohols using very long-chain fatty acid precursors as substrates. In a *FAR1* mutant of *A. thaliana*, the amount of C22:0–OH was reduced by 30% in roots and seed coats [47]. None of the seven tobacco *FAR* genes were expressed in GLTs, perhaps because 1-docosanol cannot be detected in GLTs. In addition, several *CER* genes were either not or lowly expressed in GLTs. The encoded enzymes catalyze reactions involved in long-chain alkane biosynthesis. Suberin is a lipid-phenolic biopolyester containing a variety of C16–C24 chain-length aliphatics and high amounts of glycerol and phenolics, especially ferulic acid. The products of the phenylpropanoid biosynthesis pathway supply the substrate in suberin biosynthesis. The *AtHHT1* gene encodes a feruloyl transferase that catalyzes the acyl transfer from feruloyl-coenzyme A to ω-hydroxyfatty acids and fatty alcohols [48]. Six of seven *HHT1* genes were up-regulated at the transcript level in GTs (Fig. 4), which resulted in the increased accumulation of suberin in GTs.

Tobacco trichomes excrete flavonoids, such as quercetin and methylated derivatives, after elicitation by methylJA, herbivore attack, or UV-C exposure [49, 50]. The flavonoid and isoflavonoid biosynthesis pathway are significantly enriched for multiple gene families. The biosynthesis of flavonoids in plants originates from phenylpropane compounds, and isoflavonoids are synthesized from flavonoids. The expression levels of multiple genes in this pathway were down-regulated, which may reduce the contents of multiple compounds in GLTs. Thus, we concluded that the GTs strongly affect the levels of flavonoid and isoflavonoid biosynthesis.

In addition, the differences between GLTs and GTs were reflected in photosynthesis. The normal chloroplasts in *N. tabacum* trichomes are necessary for a photosynthetic capability to synthesize certain exudate compounds. For example, multiple *LHCB1* homologous genes relating to photosynthesis are only expressed in GTs, which have a multicellular gland at the top. The development of chloroplasts in head cells of GTs may cause the biosynthesis of terpene compounds [51, 52].

The transcription factors families, such as R2R3-MYB family proteins, C2H2 zinc finger proteins, bHLH type proteins, and HD-ZIP type proteins, play an important role in plant trichomes development [20]. The R2R3-MYB subfamily is closely involved in the development of trichomes, especially in Arabidopsis, and MYB can form a complex with TRANSPARENT TESTA GLABRA1 and bHLH proteins to participate in trichome formation [53, 54]. In the study, the overexpression of a MIXTA-like gene *Nitab4.5_0011760g0030.1* resulted in an increase in the number of branched trichomes in tobacco. In Arabidopsis, MIXTA gene AtMYB106 negatively regulates the number of trichome branches [55]. Nevertheless, the SlMIXTA1 and SlMixta-like control trichome type and density in tomato [21, 22]. Although these genes belong to the MIXTA gene subfamily, they have divergent functions in trichomes development. In *Nicotiana benthamiana*, NbMYB123-like positively regulates glandular trichomes density by acting downstream of NbGIS [56]. The results of RNA-Seq analysis revealed that three MYB transcription factors showed higher expression in GLT compared to GT and other tissues. On the contrary, one member was almost exclusively expressed in GT. Nevertheless, further experimental validation is needed to determine whether these genes are also involved in the development of glandular trichomes.

Nitab4.5_0000091g0520.1 is the HD-ZIP IV transcription factor with the higher homology to tomato SlWo. The gene has higher expression level in GT than in GLT. The *SlWo* gene regulates tomato type I trichome formation mainly through heterodimer formation with the B-type cyclin SlCycB2 [57]. In *N. benthamiana*, Nbwo is the homologous to SlWo and can be combined with the NbCycB2 promoter sequence [58]. As a form of feedback regulation, NbCycB2 inhibits Nbwo activity to negatively regulate trichome formation. In our data, two homologous to Nbwo with highest sequence similarity show no differential expression in GT and GLT. They are also expressed in other tissues that possess trichomes, including flowers, stems, leaves. Two HD-ZIP IV genes, *Nitab4.5_0002049g0010.1* and *Nitab4.5_0000143g0600.1*, were found to be highly similar to the Arabidopsis homeodomain GLABROUS 2, which is closely involved in the development and initiation of trichomes [59]. These two genes exhibited high expression levels in GTs, but they were expressed at low levels or not expressed at all in GLTs and other tissues. It is worth conducting further research on whether these two genes are related to glandular trichomes development.

The C2H2 transcription factor *NbGIS* positive regulated tobacco glandular trichome initiation through GA signaling [56]. The expression levels of the two orthologs of NbGIS are very low, but their expression levels in GLT are higher than in GT. In contrast, one orthologs of tomato C2H2 transcription factor SIZFP6 is more highly expressed in GT than in GLT. Overexpression of *SlZFP6* led to an increase in the density of type I, III/V, VI, and VII trichomes, as well as the length of type I, III, and VI trichomes in tomato [30]. Additionally, C2H2 zinc finger proteins H interacts with SlZFP8L to regulate the initiation and elongation of tomato trichome by modulating *SlZFP6* expression [30]. Among the 12 differentially expressed C2H2 transcription factors we identified, it is possible that other members are also involved in trichome development.

The bHLH transcription factor SlMYC1 is the only gene that is involved in glandular hair development without affecting the regulation of non-glandular hair. SlMYC1 regulates the development of type VI glandular hairs in tomato [31]. In addition, tomato SlbHLH95 negatively regulates tomato trichome initiation [60]. In the study, 12 bHLH transcription factors were identified that are expressed at higher levels in GLT than in GT. Further analysis is needed to determine whether there are genes specifically expressed in non-secretory glandular hairs among these genes, as the GT samples contain a low proportion of non-secretory glandular hairs. Additionally, further functional validation is required to investigate the association between these genes and glandular hair development.

In Arabidopsis, the ectopic expression of a constitutively active B-type cyclin induces mitotic divisions and increases multicellular trichome numbers [61]. In tomato, the overexpression of SlCycB2 results in a non-trichome phenotype, whereas suppression of SlCycB2 promotes trichome formation [62]. In this study, four *CycB2* genes specifically expressed in trichomes were identified in tobacco. Interestingly, three genes were highly expressed in GTs compared with in GLTs, which indicated that these *CycB2* genes might be involved in trichome development. Knocking out two *NtCycB2* can promote the formation of long stalk glandular trichomes, while overexpression of *NtCycB2* can reduce long stalk glandular trichomes density [63]. In addition, JA is a crucial signaling molecule that regulates trichome formation and development. The JAZ proteins function as transcriptional repressors of JA-responsive genes. The *SlJAZ2* suppressed glandular hair initiation by suppressing the expression of *SlWo* and *SlCycB2* in tomato [32]. The *SIJAZ4* is a negative regulator of trichome development and can interact with HD-Zip transcription factor SlHD8 [28]. In *A. annua*, AaJAZ8 can inhibit the activity of the positive regulator AaHD1 and reduce the density of glandular hairs [33]. In this study, 23 tobacco JAZ proteins have been identified, with 12 being highly expressed in trichomes. Further experimental validation is needed to determine whether the highly expressed JAZ proteins in trichomes are involved in the transduction of JA signal and the regulation of trichomes development.

So far, almost all genes identified that regulate glandular trichomes also affect non-glandular trichomes development in plants except *SIMYC1*. Therefore, it is necessary to further explore the genes that independently regulate glandular trichomes development. The application of advanced technology, such as microdissection technology, omics technology, single-cell sequencing and CRISPR-Cas9, will contribute to future research on the molecular mechanisms of plant trichomes development.

## Conclusion

In summary, this study obtained 2,828 DEGs by comparing the transcriptome of GLTs with that of GTs. Enrichment analyses of the DEGs revealed that their main functions related to cutin, suberine, and wax biosynthesis, flavonoid and isoflavonoid biosynthesis, terpenoid biosynthesis, and plant-pathogen interaction, with the DEGs being up-regulated in GTs. This may increase the contents of secreted compounds in glandular trichomes. In addition, numerous transcription factor and genes that highly expressed in trichomes or specially expressed in GT or GLT were identified. Further experimental validation is necessary to determine whether these genes are involved in glandular trichome development. These results provide a molecular basis for the study of glandular trichomes development and secondary metabolism.

### Supplementary Information

Supplementary Material 1.

## Data Availability

Sequence data that support the findings of this study have been deposited in the NCBI with the primary accession code PRJNA786372.

## Abbreviations

GT: Glandular trichome
GLT: Glandless trichome
qRT-PCR: Quantitative real-time PCR analysis
DEG: Differentially expressed gene
GO: Gene ontology
KEGG: Kyoto Encyclopedia for Genes and Genomes
