# Supplementary material for: Comparative transcriptome analysis reveals genes involved in trichome development and metabolism in tobacco
Source: BMC Plant Biol. 2024 Jun 13;24:541. doi: 10.1186/s12870-024-05265-4 (PMC11177470; doi:10.1186/s12870-024-05265-4)
Supplement: Supplementary file 1 — Supplementary Material 1. [file 12870_2024_5265_MOESM1_ESM.docx]

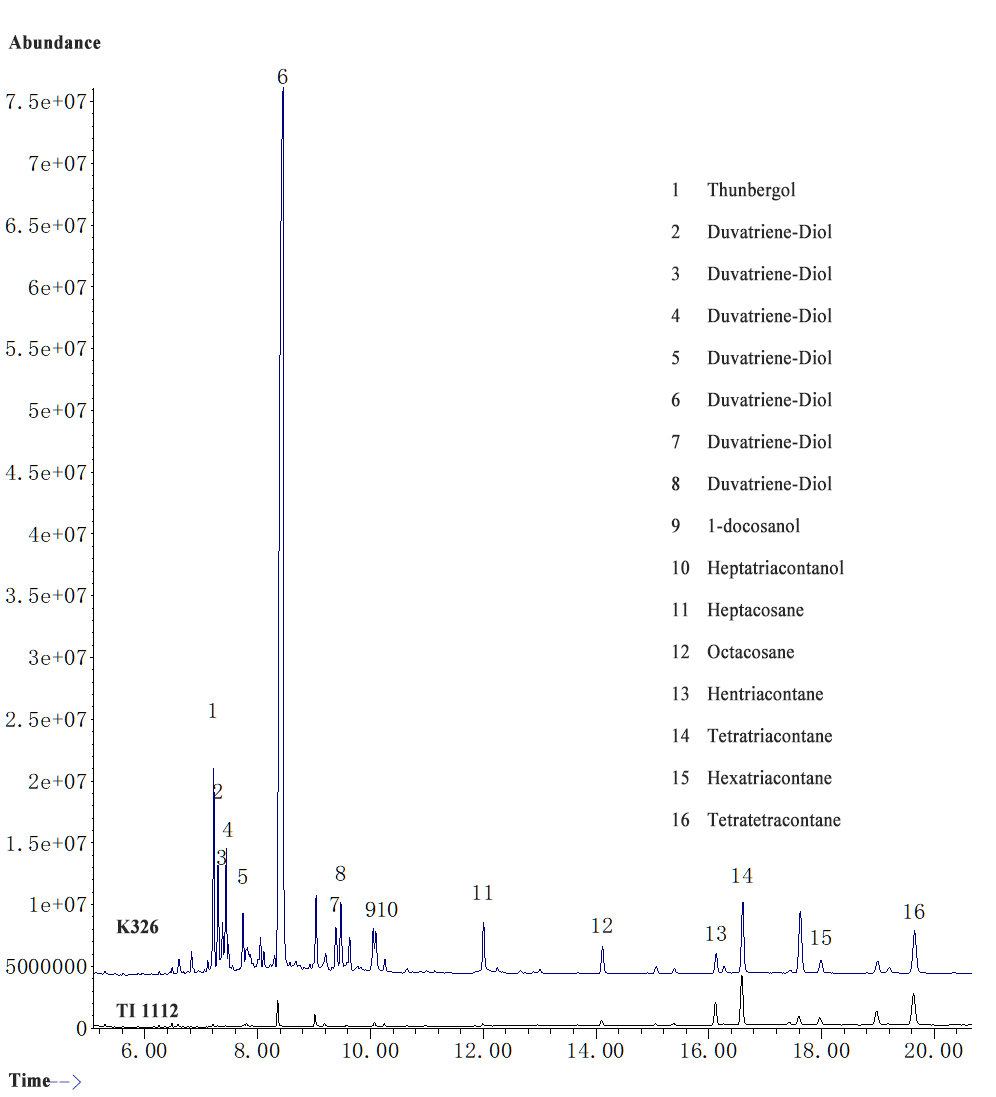
Figure S1. GC-MS of the cuticular chemicals of K326 and TI 1112

Table S1 Summary of RNA-Seq reads for GT and GLT

| **sample** | **clean_reads** | **clean_bases** | **Q30** | **GC_pct** |
| --- | --- | --- | --- | --- |
| GLT_1 | 46070340 | 6.91G | 92.71 | 44.73 |
| GLT_2 | 44118652 | 6.62G | 92.41 | 44.78 |
| GLT_3 | 45137878 | 6.77G | 93.03 | 44.96 |
| GT_1 | 49298256 | 7.39G | 91.93 | 43.79 |
| GT_2 | 43944310 | 6.59G | 92.13 | 43.45 |
| GT_3 | 46706558 | 7.01G | 92.24 | 43.45 |

Table S2. The primers used for qRT-PCR

| Gene ID | Annotation | Forward Primer | Reverse Primer |
| --- | --- | --- | --- |
| Nitab4.5_0009320g0010.1 | Actin | CAAGGAAATCACCGCTTTGG | AAGGGATGCGAGGATGGA |
| Nitab4.5_0004287g0090.1 | LytB protein | TGTGATGCAACTCAAGAGCG | ACTCACCATGCAGCAACTTG |
| Nitab4.5_0000842g0120.1 | Myb-related protein | GGCAGGGTTCGAGTTCTAGT | TTGGCTTGCTCTCCTCTTGA |
| Nitab4.5_0012814g0010.1 | 4-hydroxy-3-methylbut-2-en-1-yl diphosphate synthase | ACGGAAGGATGAAGTCTGCA | TTCTTCAAAAGCGGCCACTC |
| Nitab4.5_0002300g0020.1 | 4-diphosphocytidyl-2-C-methyl-D-erythritol kinase | TTGGTGGTGGGAGCAGTAAT | AATGGTATGGGCGATGGGAT |
| Nitab4.5_0000402g0040.1 | 1-deoxy-D-xylulose-5-phosphate synthase | TGGCTCCATCAGATGAAGCA | CTGCAGCTCCCAAACATTGT |
| Nitab4.5_0004704g0020.1 | Myb-related protein | AAGCCGAATTTGCCGAATCT | AAACAAGTCAGCAGCATCGG |
| Nitab4.5_0000889g0030.1 | Myb-related protein | GGACGAGGTTGTTGCTTTCA | CCATGCCTCTTCCAATTCCC |
| Nitab4.5_0007274g0030.1 | Homeobox-leucine zipper protein | CCTGCCACCACTCTCACTAT | GTGCAGGTGGAAATGGGAAG |
| Nitab4.5_0000697g0100.1 | Protein ECERIFERUM | CTGAGCCCAAAGTTGTGACC | CCACGTCCGATACTCCATGA |
| Nitab4.5_0001375g0050.1 | GDSL esterase/lipase | TGGTAGCATGTTGTGGAGGT | TGGAGAAGTGAAAGGGCCAT |
| Nitab4.5_0010777g0020.1 | Pectinesterase | GTGCAGCTCCAACTTCAAGT | GAAAGTAGTCCACCCGTCGA |
| Nitab4.5_0000592g0350.1 | Chlorophyll a-b binding protein | AAGTTTGGTCCACGCACAAA | CAAGGCCCAATGGATCGAAG |
| Nitab4.5_0002814g0030.1 | Chlorophyll a-b binding protein | GATTTTCAGCGAGGGTGGTC | CCTTTACCTTGAGCTCAGCG |
| Nitab4.5_0000863g0150.1 | ABC transporter G family member | GGCTCAGCTGGAAGAAAACC | TGCGTAGTGAAGTTGGCATG |
| Nitab4.5_0001103g0080.1 | Secoisolariciresinol dehydrogenase | CGGCCAAGAAAGGTGTCATT | CCTCCTGCTGCAAACCATTT |
| Nitab4.5_0011760g0030.1 | Myb-related protein | TTCTGCGCATGTGGAACTCA | ACCATCAACAACACCGAGCT |
